# Surface Plasmon Resonance, Formation Mechanism, and Surface Enhanced Raman Spectroscopy of Ag^+^-Stained Gold Nanoparticles

**DOI:** 10.3389/fchem.2019.00027

**Published:** 2019-02-14

**Authors:** Sumudu Athukorale, Xue Leng, Joanna Xiuzhu Xu, Y. Randika Perera, Nicholas C. Fitzkee, Dongmao Zhang

**Affiliations:** ^1^Department of Chemistry, Mississippi State University, Starkville, MS, United States; ^2^Department of Chemistry, Chengdu University of Technology, Chengdu, China; ^3^Department of Chemistry, Xihua University, Chengdu, China

**Keywords:** anti-galvanic reaction (AGR), gold nanoparticles, silver, zeta potential, Raman

## Abstract

A series of recent works have demonstrated the spontaneous Ag^+^ adsorption onto gold surfaces. However, a mechanistic understanding of the Ag^+^ interactions with gold has been controversial. Reported herein is a systematic study of the Ag^+^ binding to AuNPs using several *in-situ* and *ex-situ* measurement techniques. The time-resolved UV-vis measurements of the AuNP surface plasmonic resonance revealed that the silver adsorption proceeds through two parallel pseudo-first order processes with a time constant of 16(±2) and 1,000(±35) s, respectively. About 95% of the Ag^+^ adsorption proceeds through the fast adsorption process. The *in-situ* zeta potential data indicated that this fast Ag^+^ adsorption is driven primarily by the long-range electrostatic forces that lead to AuNP charge neutralization, while the time-dependent pH data shows that the slow Ag^+^ binding process involves proton-releasing reactions that must be driven by near-range interactions. These experimental data, together with the *ex-situ* XPS measurement indicates that adsorbed silver remains cationic, but not as a charged-neutral silver atom proposed by the anti-galvanic reaction mechanism. The surface-enhanced Raman activities of the Ag^+^-stained AuNPs are slightly higher than that for AuNPs, but significantly lower than that for the silver nanoparticles (AgNPs). The SERS feature of the ligands on the Ag^+^-stained AuNPs can differ from that on both AuNPs and AgNPs. Besides the new insights to formation mechanism, properties, and applications of the Ag^+^-stained AuNPs, the experimental methodology presented in this work can also be important for studying nanoparticle interfacial interactions.

## Introduction

Gold nanoparticles (AuNPs) are among the most studied nanomaterial owing to their unique photochemical properties and their applications in biosensing, catalysis, biomedicine, electronics, and surface-enhanced Raman spectroscopy (SERS) (El-Sayed et al., 2006; Anker et al., 2008; Ghosh et al., 2008; Brown et al., 2010; Homberger and Simon, 2010; Zhou et al., 2010; Ansar et al., 2011; Athukorale et al., 2018). Current methods for preparing AuNPs with different structural characteristics and functionalities can be categorized into two classes of approaches. The first class is by synthetically controlling AuNP sizes, shapes, and chemical compositions (Jana et al., 2001a,b; Bastús et al., 2011) and the second is to tune the AuNP structural parameters and functionality through post-modification of AuNP surface (Daniel and Astruc, 2004; Eustis and El-Sayed, 2006; Dykman and Khlebtsov, 2012; Siriwardana et al., 2014). Indeed, AuNP surface chemistries, including ligand interfacial interactions of plasmonic AuNPs, have evolved as one of the most active research areas due to their importance in essentially every aspect of AuNP applications (Perera et al., 2016a,b; Athukorale et al., 2018). The most common ligands used for AuNP surface functionalization are proteins (Vangala et al., 2012; Siriwardana et al., 2013; Wang et al., 2016; Woods et al., 2016), organothiols (Ansar et al., 2011, 2013b, 2014), and thiolated chemicals such as poly(ethylene glycol) (Siriwardana et al., 2014).

Silver binding to gold has been the focus of several recent publications (Wu, 2012; Wang et al., 2014; Kang et al., 2015; Athukorale et al., 2017a; Liu and Astruc, 2017; Nguyen et al., 2018). However, most of the gold substrates are aggregated AuNPs or planar gold films including gold electrodes. The mechanism of binding has been ambiguous, too. Primarily on the basis of x-ray photon spectroscopy (XPS) analysis, Wu et al. proposed that Ag^+^ binding to AuNP proceeds through an anti-galvanic reduction in which the Ag^+^ is reduced into Ag^0^ by the chemically inert Au surface atom (Wu, 2012; Wang et al., 2014). However, the reliability of the XPS for identification of the Ag charge state is highly questionable. Indeed, a survey of the National Institute of Standards and Technology (NIST) X-ray photoelectron spectroscopy database shows that the binding energy of 3d(Wu, 2012)_3/2_ electron for the zero valence silver varies from 373.40 to 374.27 eV, while that for Ag^+^ 3d_3/2_ is 373.90 eV (Naumkin et al., 2012). The binding energy of the 3d_5/2_ electron for the zero valence silver is from 367.90 to 368.40 eV, while that for the Ag^+^ is from 367.40 to 369.00 eV (Naumkin et al., 2012). The fact that Ag^0^ and Ag^+^ overlap tremendously in both their 3d_3/2_ and 3d_5/2_ electron binding energy indicates the difficulty of unambiguously assigning silver charge state using XPS data.

Care should be exercised on XPS data interpretation. Literature XPS analyses were performed almost exclusively with dried samples (Wu, 2012; Wang et al., 2014; Perera et al., 2018). However, the sample drying process can change the ligand surface composition through multiple mechanisms. As an example, as-synthesized AuNPs are usually negatively charged, but the dried AuNPs are charge-neutral. Such charge neutralization can occur either by reducing the number of the anionic species on the AuNPs and/or by enhancing adsorption of the cationic species that are initially confined within the electrical double layer or in the bulk solution. Furthermore, solvent drying also introduces the adsorption of impurities onto AuNP surfaces, further compromising the reliability of the measurement results (Perera et al., 2018). Exposing the XPS samples to ambient air also introduces impurities. Indeed, even a brief exposure of X-ray cleaned gold film into air or Nanopure water can introduce significant surface contamination. Such surface contamination can be readily concluded by the appearance of carbon and oxygen species in the XPS spectrum obtained with gold film (Perera et al., 2018). Unfortunately, Wu et al. have shown the XPS features for silver and gold alone, with no information given for other elements in their publications, making it impossible to assess the complexity of contaminating surface adsorbates on their AuNPs (Wu, 2012; Wang et al., 2014).

Indeed, electrolyte binding to AuNPs should be studied with measurement strategies capable of differentiating ionic species directly absorbed on the surface and those confined within the electrolyte double layer or diffuse in the bulk solution. We recently reported the spontaneous adsorption of Ag^+^ onto all explored gold substrates, including pure gold foil, sputter-coated gold film, and washed AuNP aggregates synthesized with both citrate- and borohydride reaction methods (Athukorale et al., 2017a). The adsorbed silver most likely remains cationic as a component of the insoluble salts attached onto the AuNP surfaces. The key supporting evidence is: (1) There are carbon and oxygen features in these samples whenever silver XPS peaks are observed, (2) A drastic reduction of solution pH reduction accompanies spontaneous Ag^+^ adsorption, a phenomenon that can't be explained by the proposed anti-galvanic mechanism, (3) Ag^+^ induces desorption of the soluble organothiols that are initially anchored onto the AuNPs.

Herein we provide further supporting evidence that Ag^+^ adsorbed onto gold surface remains as a cation and not as a charge-neutral silver atom. In this study, we use the dialysis-purified and citrate-reduced AuNPs, instead of the AuNP aggregates or planar gold film as the model gold substrate. The dialyzed AuNPs maintain their dispersion stability in solution, as does the monolayer Ag^+^-functionalized AuNPs. This enabled us to investigate the effect of Ag^+^-staining on the AuNP charge states through zeta-potential titration and time-dependent zeta potential measurement. Further, by taking advantage of the fact the Ag^+^ adsorption introduces significant spectral change in the AuNP surface plasmonic resonance, we probed the kinetics of the Ag^+^ adsorption using the time-resolved UV-vis spectroscopic analysis. The structure and properties of the Ag^+^-treated AuNPs were also studied with surface enhanced Raman spectroscopy (SERS) by using butanethiol (BuT), a monothiol, and 1,4-benzenedimethanethiol (BDMT), a dithiol as the model probes, which provides rich information regarding the mobility of the surface silver and the SERS activity of the Ag^+^-stained AuNPs.

## Materials and Methods

### Materials and Equipments

All the chemicals were purchased from Sigma-Aldrich and used as received. Nanopure water (18 MΩ-cm) (Thermo Scientific) is used throughout all experiments. UV-vis spectra were acquired with an Olis HP 8452 diode array spectrophotometer. Polarized resonance synchronous spectra (PRS2) were acquired using the Fluoromax-4 spectrofluorometer equipped with an excitation and detection polarizer. PerkinElmer ELAN DRC II inductively coupled plasma-mass spectrometer (ICP-MS) was used for the silver quantification. Litesizer 500 (Anton-Paar) instrument was used for the zeta potential measurements. A Thermo Scientific K-Alpha X-ray photoelectron spectrometer system was used for the XPS measurements. A LabRam HR800 confocal Raman microscope was used for Raman and SERS acquisitions with 632 nm laser excitation. Reflective sample substrate (RSS) slides from Raminescent, LLC were used for all SERS acquisitions. These RSS slides are highly reflective with negligible fluorescence and Raman background (Athukorale et al., 2017a).

### AuNP and AgNP Synthesis

Aqueous ~13 nm (diameter) AuNP were synthesized in-house using the citrate reduction method (Frens, 1973). The ~30 and ~50 nm AuNPs were synthesized according to the reported kinetically controlled citrate-reduced method described by Bastús et al. (2011). The size and concentration of the as-synthesized AuNPs were estimated on the basis of the AuNP UV-vis extinction spectra (Hurst et al., 2006; Zhao et al., 2008). AgNPs were synthesized in-house using the Lee-Meisel method (Lee and Meisel, 1982). Briefly, 0.027 g of AgNO_3_ was dissolved in 150 mL of Nanopure water, and the solution was heated. Then 3 mL of 1% trisodium citrate dehydrate was added when the solution starts to boil, and the mixture was kept boiling for ~60 min with stirring.

### AuNP Silver-Staining

All the as-synthesized AuNPs were dialyzed four times with Nanopure water using standard regenerated-cellulose membrane (MWCO is 12–14 kD) purchased from Spectrum Labs. The as-received dialysis membranes were washed thoroughly before use. The silver staining was conducted by adding known amount of AgNO_3_ to the dialyzed AuNPs and mixed using a vortex mixer. The mixture was left to sit overnight at ambient conditions before further usage. The kinetics of the Ag^+^ binding to AuNP was conducted with the time-resolved UV-measurements for the sample prepared by mixing the dialysis-purified 13 nm AuNPs.

### PRS2 Acquisition and Analysis

PRS2 is a new spectroscopic method that enables quantitative decomposition of the sample UV-vis extinction spectrum into its absorption extinction and scattering extinction spectrum. The theoretical background, spectral acquisition, and data analysis procedure are available in recent publications (Athukorale et al., 2017b; Siriwardana et al., 2017; Xu et al., 2018a,b). Briefly, the excitation polarization of the spectrofluorometer is set vertical (V) and the detection polarization is kept either vertical or horizontal (H) to acquire PRS2 VV or VH spectra, respectively. The depolarization is, by definition, the ratio of VH and VV intensity corrected by the polarization bias G factor. The as-acquired PRS2 solution spectra were inner-filter-effect (IFE) corrected and solvent background removed to get the analyte specific PRS2 spectra for further data analysis. The slit width of the excitation and detection monochromator is set as 2 nm with wavelength increment of 1 nm. The integration time for each wavelength variable is 0.3 s. The G-factor spectrum for correcting the instrument polarization bias, the effective pathlength for correction the sample inner filter effect, and cuvette and solvent PRS2 spectra for background subtraction were obtained using the method described previously (Siriwardana et al., 2017).

### Zeta-Potential Measurements

Dialyzed AuNPs were mixed with AgNO_3_ of predefined concentrations and incubated overnight before performing Ag^+^-concentration-dependent zeta potential measurements. The time-resolved AuNP zeta potentials were acquired as a function of the Ag^+^ treatment time for a freshly prepared AuNP/AgNO_3_ mixture. All zeta potentials were measured at 25°C in a reusable 350 μL Omega Cuvette Z (Anton-Paar). Each zeta potential is an average of 500 scans. The sample solution was equilibrated in the cuvette for 2 min prior to each acquisition.

### SERS Spectral Acquisitions

All the SERS spectra were acquired using Olympus 10 × objective (NA = 0.25). The He/Ne laser with an excitation wavelength of 632 nm was used and the laser power before the objective was 1.3 mW for the SERS acquisition. The spectrograph grating was 600 grooves/mm. The acquisition times for SERS spectra were varied between 10 and 200 s. The Raman shift was calibrated with a neon lamp.

### ICP-MS Quantification for the Adsorption of Silver on to AuNPs

The amount of Ag^+^ adsorbed was quantified using similar procedure described previously (Athukorale et al., 2017a). The AuNPs/AgNO_3_ mixture was incubated overnight and centrifuged at 9,000 rcf for 1 h so that the AuNP-containing pallet and supernatant were separated. In order to quantify the adsorbed Ag^+^, AuNP-containing pallet was centrifuged and washed several times to remove the free Ag^+^ ions and a known volume of freshly prepared aqua regia was added to digest the sample (Note that aqua regia is very corrosive and must be handled with extreme caution). A known volume of the supernatant was digested with aqua-regia to quantify the free Ag^+^ ions in the solution. All the digested samples were diluted with 18 MΩ-cm Nanopure water.

## Results and Discussion

### Effect of Silver-Staining on AuNP Surface Plasmon Resonance

As-synthesized AuNPs are purified by dialysis prior to the silver-staining, in order to remove the excess chloride, citrate, and other by-products in the AuNP synthesis solution. This is because Ag^+^ can react with the excess reagents, complicating the study of the Ag^+^ binding to AuNPs. While the dialysis is effective in removal of the free small molecules in solutions (Figure S1), it is inadequate to eliminate the surface-bound citrate and the adsorbed impurities as we demonstrated earlier with AuNPs synthesized with deuterated citrate (Perera et al., 2018). Further evidence indicating the presence of surface adsorbates on the dialyzed AuNPs comes from zeta potential measurements (see below).

**Figure 1:**
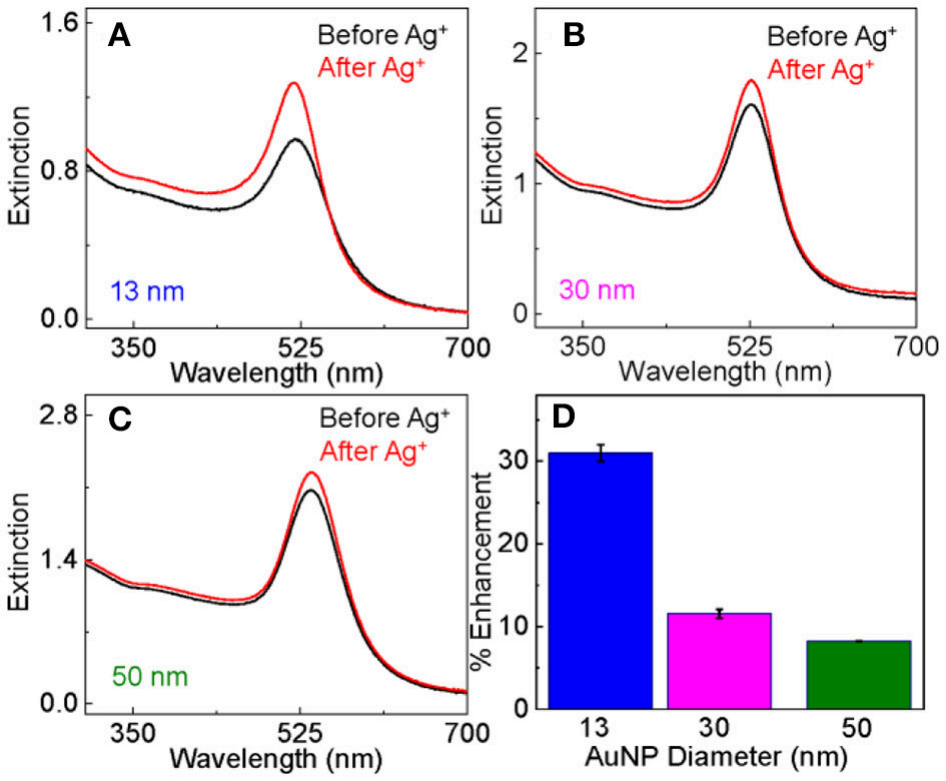
Comparison of the UV-vis extinction spectra of as-prepared dialyzed AuNPs before and after the Ag^+^-staining for AuNPs with particle size of **(A)** 13 nm, **(B)** 30 nm, and **(C)** 50 nm. **(D)** Bar plot showing the percentage increase in the AuNP peak UV-vis intensity induced by Ag^+^-staining as a function of AuNPs size.

Ag^+^ treatment of the dialyzed AuNPs induces significant change in the AuNP surface plasmonic resonance. The UV-vis peak intensity increased by 30 ± 3, 12 ± 1, and 8 ± 1% after the Ag^+^ treatment for the AuNPs with particle size of 13, 30, and 50 nm, respectively (Figure 1). The fact that the relative change in the AuNP surface plasmon resonance intensity decreases with increasing AuNP particle sizes is expected because the Ag^+^ adsorption perturbs the structure of the immediate AuNP surface layer. This is because the surface to volume ratio decreases with increasing particle sizes.

**Figure 2:**
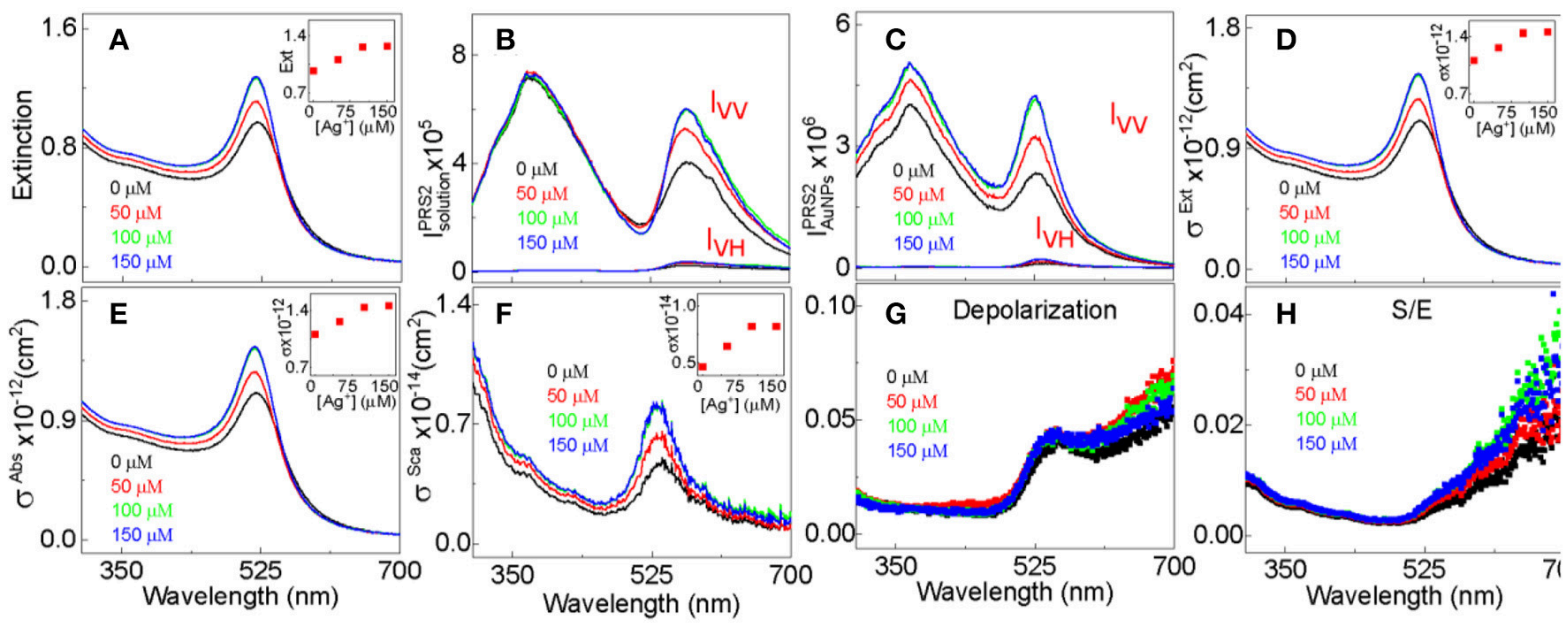
PRS2 quantification of the extinction, absorption, and scattering cross-section, depolarization, and S/E for 13 nm- AuNPs stained with 50, 100, and 150 μM AgNO_3_. An example data analysis of PRS2 spectra are showed in the Figure S2. **(A)** UV-vis extinction spectra, **(B)** as-acquired PRS2 spectra, **(C)** sample inner-filter-effect- and solvent-background-corrected PRS2 spectra, **(D–F)** extinction, absorption, and scattering cross-section spectra, respectively. **(G,H)** The AuNP depolarization spectra and S/E spectra, respectively. The Ag^+^ concentration for the spectrum in black, red, green, and blue are 0, 50, 100, and 150 μM, respectively. Insets in **(A,D–F)** showing the spectral peak intensity as a function of the Ag^+^ concentration.

UV-vis evaluates the sample total photon extinction, the sum of the photon absorption extinction and scattering extinction. With sample UV-vis (Figure 2A) and PRS2 spectra (Figures 2B,C), we decomposed the AuNP UV-vis extinction cross-section spectra (Figure 2D) into their absorption and scattering component spectra (Figures 2E,F), and quantified the AuNP light scattering depolarization spectrum (Figure 2G) as well as the scattering to extinction ratio spectrum. This enables us to reveal insights that have not been accessible before. We have recently demonstrated that light scattering depolarization is very sensitive to the deformation of the spherical AuNPs (Xu et al., 2018a). The silver-staining has no significant effect on the AuNPs light scattering depolarization spectrum, indicating that the AuNPs remain spherical after the silver-staining. The fact that Ag^+^ adsorption increases both the absorption and scattering extinction intensity (Figures 2D,E), but not the scattering to extinction ratio (S/E) spectrum intensity (Figure 2H) indicates that silver-staining enhanced the photon absorption and scattering by approximately the same degree.

### Kinetics and Adsorption Capacity on Ag^+^ Adsorption

**Figure 3:**
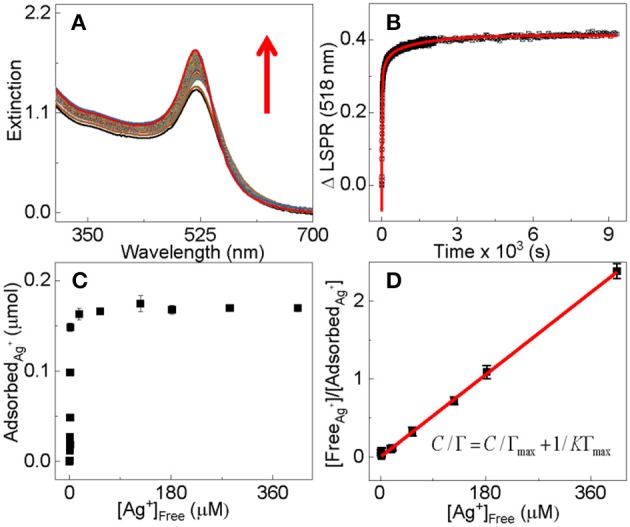
**(A)** Time-resolved UV-vis extinction spectra for the adsorption of Ag^+^ onto dialyzed AuNPs of 13 nm in diameter, **(B)** time-course for the AuNP UV-vis intensity change at 518 nm in the spectra shown in **(A)**. Black dots are experimental data and the red solid curve is the fitted curve with the two pseudo-first-order reaction equation (Equation 1). The nominal concentration of Ag^+^ is 100 μM for the time-resolved UV-vis data. **(C)** ICP-MS quantification of the Ag^+^ adsorption onto dialyzed AuNPs as a function of the concentration of the excess Ag^+^. The samples are prepared by mixing 1 mL of the dialyzed 13 nm AuNPs with equal volume of AgNO_3_. The nominal Ag^+^ concentrations in the mixtures are 1, 6, 12, 20, 50, 100, 150, 180, 200, 220, 300, and 575 μM, respectively. **(D)** Empirical Langmuir fitting of the adsorption data in **(C)**.

The fact that the Ag^+^ adsorption induced a significant change to the AuNP surface plasmon resonance spectrum allows us to study the adsorption kinetics using time-resolved UV-vis measurements (Figure 3A). The working hypothesis is that the change in the AuNP surface plasmon is proportional to the amount of adsorbed Ag^+^ (Figure 3B). Over 50% of the silver adsorption occurs within the measurement's 10 s dead time in the time-resolved UV-vis study (Figure 3B). This is extraordinarily fast considering the ultralow AuNP concentration (low nM) and its small mobility in solution as a nanoparticle. Fitting the time-resolved UV-vis data with one first order equations produces large errors (Figure S3), but near perfect fitting is achieved with two pseudo-first-order reaction equations that yield reaction time constants of τ_1_ = 16 ± 2 s and τ_2_ = 1,000 ± 35 s, respectively. Therefore, the Ag^+^ adsorption process can be approximated empirically by two parallel first-order reactions differing drastically in the reaction time scale. The Γ_1_ and Γ_2_ values associated with the two time constants are 0.4 and 0.079, respectively, indicating that over 94% of the Ag^+^ adsorption occurs through the fast process that has the time constant of 16(±2) s.

(1) $$\begin{array}{l} M=\Gamma_{1\;}\left(1-exp\left(-t/\tau_{1}\right)\right)+\Gamma_{2\;}\left(1-exp\left(-t/\tau_{2}\right)\right) \end{array}$$

Empirically, the Ag^+^ adsorption onto dialyzed AuNPs follows a Langmuir adsorption isotherm with the binding constant and saturation packing density of K~ 4 × 10^6^ M^−1^ and Γ_max_~4.7 nmol/cm^2^, respectively (Figures 3C,D). The saturation packing density of Ag^+^ on AuNPs was calculated similarly as before (Athukorale et al., 2017a) by assuming the AuNPs are perfectly spherical with identical sizes. The binding constant and packing density of the Ag^+^ on the dialyzed AuNP are higher than their respective counterparts on the extensively washed citrate-reduced AuNPs that are pre-aggregated with KNO_3_. The binding constant and packing density for the Ag^+^ adsorption onto pre-aggregated AuNPs are 4.3 × 10^3^ M^−1^ and ~2.8 nmol/cm^2^ respectively (Athukorale et al., 2017a). The small difference in the Ag^+^ packing density between the dialyzed AuNPs and the pre-aggregated AuNPs can be readily understood by the fact that the pre-aggregated AuNPs must have a smaller fraction of surface area accessible for Ag^+^ adsorption.

**Figure 4:**
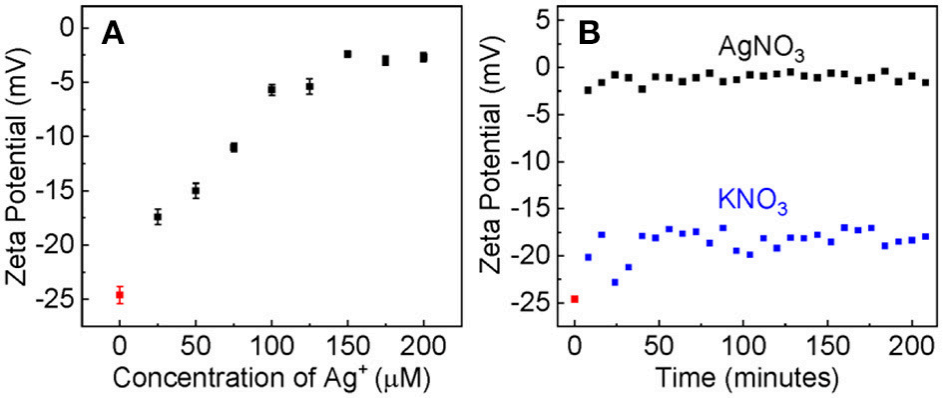
**(A)** The AuNP zeta potential as the function of the nominal Ag^+^ concentration. The AuNP/AgNO_3_ mixtures were incubated overnight before measurements. **(B)** The time-dependent zeta potential of AuNPs treated with AgNO_3_ or KNO_3_ with the same nominal concentration of 100 μM.

The large difference in the Ag^+^ binding constant between the dialyzed and pre-aggregated AuNPs is due most likely to the difference in the surface charge states as well as the chemical compositions on the AuNPs that are caused by the post-synthesis process. Zeta-potential measurement reveals that the dialyzed AuNPs remain highly negatively charged (Figure 4), but the charge density on the aggregated AuNPs should be likely negligibly small. Therefore, due to the electrostatic attraction, the Ag^+^ adsorption onto the dialyzed AuNPs is energetically more favorable than that onto the pre-aggregated AuNPs.

Evidence supporting that the electrostatic interaction comes from both the concentration-dependent and time-resolved zeta potential measurements (Figure 4). Ag^+^ adsorption neutralizes the surface charge of the dialyzed AuNPs. The AuNP zeta potential monotonically increases (becomes less negative) with increasing Ag^+^ concentration before it reaches a plateau value of 3 mV when the nominal Ag^+^ concentration is 150 μM (Figure 4A). This is consistent with the data obtained with UV-vis measurements that showed ~150 μM Ag^+^ induces a maximum AuNP UV-vis intensity change (Figure 1). Moreover, the Ag^+^-induced charge neutralization of the AuNPs is a very rapid process, completed within the 8 min dead time of the zeta potential measurement (Figure 4B).

**Figure 5:**
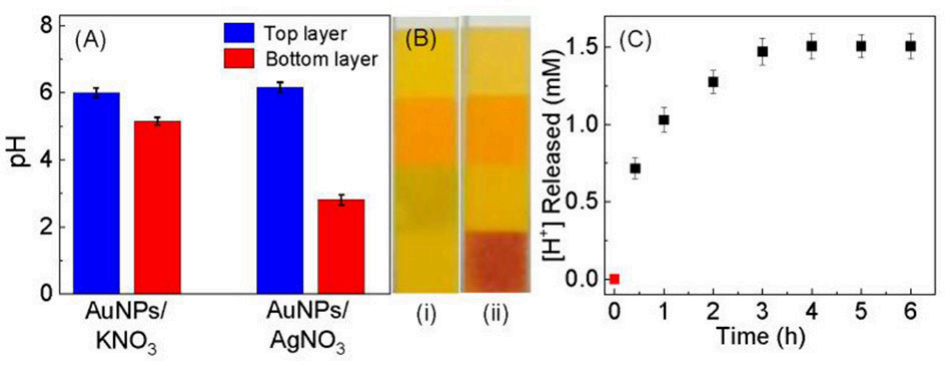
**(A)** Bar plot showing the pH change occurring in solution after the addition of KNO_3_ and AgNO_3_. 30 mL of dialyzed AuNPs was centrifugation precipitated and split into two equal volume portions. Those two are AuNP-free top layer and AuNP-containing bottom layer. pH measurements were conducted 1 h after incubating top and bottom layer with 10 mM KNO_3_ and AgNO_3_. **(B)** Photograph of the pH papers used to check the pH of the Ag^+^ treated (i) top and (ii) bottom layers, respectively. **(C)** Time-dependent proton concentration as a function of the (AgNO_3_/AuNP) incubation time. The sample was prepared by mixing 10 mM of AgNO_3_ with 30 mL of dialyzed AuNPs that were centrifugation precipitated before AgNO_3_ addition.

Like what has been observed in the Ag^+^ binding to other gold substrates, including the high-purity gold foil, planar gold film, and aggregated AuNPs (Athukorale et al., 2017a), Ag^+^ adsorption on the dialyzed AuNPs also induces significant pH change in the AgNO_3_/AuNP solution (Figure 5). Kinetically, however, the rate of this proton generation reaction is drastically slower than the Ag^+^-adsorption-induced zeta potential change that occurs instantaneously upon AgNO_3_ addition. It takes more than 2 h for the solution pH or proton concentration to reach a constant value. Beside the intrinsic proton generating reaction as it will be further discussed, slow mass-transfer in the pH measurement samples can also contribute to the slow proton releasing induced by the AgNO_3_ addition (Figure 5C). The AuNP used in these samples were centrifugation precipitated before the AgNO3 addition (Figure 5) to prevent contamination of the pH probe by AuNPs. In contrast, the AuNPs were dispersed in solution in the UV-vis and zeta potential studies. Nonetheless, the fact AgNO_3_ are significantly more effective than KNO_3_ in causing pH reduction in AuNP-containing solution (Figure 5), confirms that the proton-release is predominantly due to the Ag^+^ adsorption.

Collectively, the time-dependent zeta potential and the pH measurement offer an excellent explanation for the empirical two-first-order-reaction model proposed on the basis of the time-resolved UV-vis study of the Ag^+^ binding to AuNPs (Figure 3). Mechanistically, the Ag^+^ binding most likely proceeds through a fast charge neutralization reaction in combination with a relatively slow proton-generating displacement reaction. This charge neutralization is driven by the long-range electrostatic force, therefore can be completed within a few minutes of the sample incubation period. This hypothesis is supported by the zeta-potential measurement. In contrast, the proton-generation reaction is most likely a displacement reaction that occurs only through interactions with proximal molecules, i.e., Ag^+^ and the ionisable-hydrogen-bearing adsorbates must be in direct contact with an appropriate orientation in order for the reaction to occur. Consequently, this displacement reaction must be slower than the Ag^+^ adsorption driven by the electrostatic interactions.

The mostly likely reason why Ag^+^ are drastically more effective than K^+^ in neutralizing the AuNP surface charge is that Ag^+^ can form insoluble particles with the surface adsorbates remaining on the dialyzed AuNPs. Earlier reports with deuterated citrate showed that citrate remains adsorbed onto AuNP surface even with extensive solvent washing (Perera et al., 2018). Chlorides in the AuNP synthesis solution can also remain on the dialyzed AuNP surfaces. The solubility of silver citrate is 5.5 × 10^−4^ mol/L (Seidell, 1928), while the solubility of AgCl is 1.2 × 10^−5^ mol/L (Seidell, 1928). In contrast, both potassium citrate and potassium chloride are highly water-soluble. Their solubility values are 5.9 mol/L and 4.8 mol/L, respectively (Seidell, 1928). Indeed, silver can readily react with citrate, forming an insoluble Ag-citrate salt (Figure S4).

The protons generated by the Ag^+^ binding are due likely to the Ag^+^ reaction with citrate or its reaction by-product that contain intact carboxylic groups. The as-synthesized AuNPs are weakly acidic with a pH value of around 5 (Gadogbe et al., 2013; Karunanayake et al., 2015). The surface bound citrates most likely bear substantial amount of the ionizable protons. The p*K*_a_ values of the three carboxylic groups of citrate acid in water are 3.2, 4.8, and 6.4 (Ji et al., 2007; Ojea-Jiménez and Campanera, 2012). However, it is unlikely all carboxylic acid groups on the surface-adsorbed citrate have been ionized under the experimental condition performed under neutral condition. First, the average p*K*_a_ values of those carboxylic groups for the citrate acid on AuNPs are likely higher, due to coulombic repulsion among confined likely-charged species (Perera et al., 2016a, p. 6392; Perera et al., 2015, p. 6245), than their respective counterpart in water. Positive pKa shifts of acids assembled onto solid substrate has been extensively reported (Sugihara et al., 2000, p. 6720; Masheter et al., 2007, p. 6721). Second, the pKa for the third carboxylic acid in citrate in water is 6.4, indicating third carboxylic group must have a substantial faction remaining intact even for citrate acid in pH ~7 solutions. The fraction of intact carboxylic acid groups in citrate adsorbed on AuNPs likely significantly higher than that dispersed in solution. While it is unreliable to experimentally measure pH due to the poor solution conductivity, pH of the dialyzed AuNPs is likely around 7.

The fact that silver has a higher saturation binding capacity on the “naked” dialyzed AuNPs (Figure 3) than that reported earlier for the organothiol- and dithiol-functionalized AuNPs also supports the proposed charge neutralization and the proton-displacing reaction pathways. The predominant surface adsorbates on the dialyzed AuNPs are likely citrate, but that on the organothiol-functionalized AuNPs are mono- or di-thiols. Early work showed that thiols can readily displace citrate on AuNP surfaces (Perera et al., 2018). Each citrate can react with three Ag^+^ through combination of the charge-neutralization and proton-displacement reactions, but mono- and di-thiol can only react with one or two Ag^+^. What further contributes to the larger Ag^+^ adsorption onto the dialyzed AuNPs than the thiol-functionalized AuNP aggregates is that all citrates in the dialyzed AuNPs are accessible for reacting with Ag^+^, but the steric hindrance in the thiol-functionalized AuNPs limits the AuNP surface accessibility. The thiol-functionalized AuNPs are all aggregated prior to the AgNO_3_ treatment (Perera et al., 2018).

### SERS of the Ag^+^-Stained AuNPs

**Figure 6:**
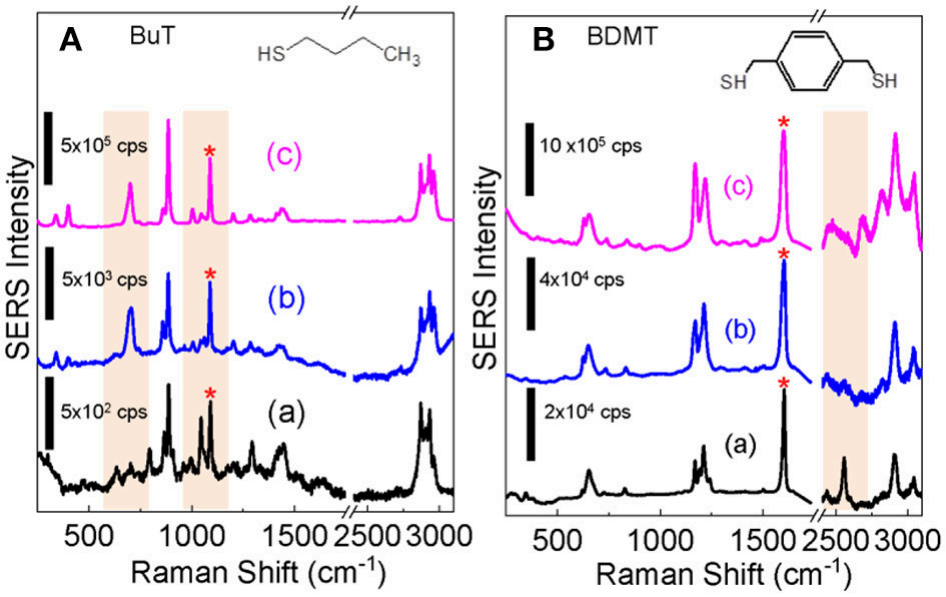
Comparison of the SERS spectra obtained for **(A)** BuT and **(B)** BDMT with dialyzed AuNPs **(a)** before and **(b)** after the Ag^+^ staining, **(c)** is the SERS spectra obtained with AgNPs. The structure of BuT and BMDT are also shown. The SERS spectra are normalized to the peak denoted by “^*^”.

Studying SERS of the Ag^+^-stained AuNPs is important not only for their potential applications as SERS substrates but also for probing the structure and properties of the Ag^+^-containing AuNPs. Taking the advantage of the excellent dispersion stability of the Ag^+^-stained AuNPs in water, we compared the SERS spectra obtained with dialyzed AuNPs, Ag^+^-stained AuNPs, and the AgNPs for both BuT and BMDT. The SERS intensity of the ligands on the Ag^+^-stained AuNPs is marginally higher than that on the as-prepared dialyzed AuNPs, but significantly lower than that on the AgNPs (Figure 6).

The structure and conformation of alkanethiols on AuNPs are deduced from its skeletal C-S and C-C stretching frequencies. The C-S stretching peaks for the *trans* υ(C-S)_*T*_, and *gauche* υ(C-S)_*G*_ conformers are at ~680 and ~610 cm^−1^, respectively and C-C stretching peaks for *trans* υ(C-C)_*T*_, and *gauche* υ(C-C)_*G*_ are ~1,070 and ~1,020 cm^−1^, respectively (Rycenga et al., 2009; Bantz et al., 2012). C-S and C-C stretching features (b) indicate that the BuT on AgNPs and Ag^+^-stained AuNPs are mostly in highly ordered *trans* conformers, but totally disordered on the AuNPs without Ag^+^ staining.

Earlier research showed that BuT is in a highly ordered *trans* conformer on the AgNPs, but totally disordered on the AuNPs (Ansar et al., 2014). This is due to the surface atoms on AgNPs are mobile, therefore the intermolecular van der Waals force among the BuT-Ag complexes drives the ordering by overcoming the constrain of the AgNP surface curvatures (Ansar et al., 2013a, 2014; Athukorale et al., 2017a). In contrast, the surface gold atoms are immobile. The BuT on the AuNPs are totally disordered due to their nanoparticle surface curvature (Ansar et al., 2014). The similarity between the BuT SERS spectra obtained with the AgNPs and Ag^+^-stained AuNP indicates that the Ag-BuT complex formed on the Ag^+^-stained AuNPs are also mobile.

Further evidence of the mobility of the surface adsorbed Ag^+^ on the Ag^+^-stained AuNPs comes from the SERS spectra obtained with dithiol BDMT. There is a relatively strong S-H peak in the 2,600 cm^−1^ region in the SERS spectrum obtained with the BDMT on AuNPs, indicating there are significant intact thiols on the BDMT on AuNPs. Earlier study has shown that the intact S-H appears only when BDMT is at upright positions when BDMT approaches full monolayer adsorption on the AuNP surfaces (Gadogbe et al., 2015). The absence of detectable S-H stretching feature in the BDMT SERS spectra obtained with the Ag^+^-stained AuNPs indicates that the adsorbed Ag^+^ on the dialyzed AuNP can reach the distal thiol that otherwise unreactive to the AuNPs.

## Conclusions

In conclusion, we have conducted a systematic study of the Ag^+^ binding to AuNPs using a series of *in-situ* techniques including the AuNP surface plasmonic resonance, zeta-potential titration, pH measurements, and SERS acquisitions. All the experimental data indicate that the Ag^+^ adsorbs onto AuNPs as cationic silver species, but not as the zero-charged silver atoms. Mechanistically, the Ag^+^ adsorption is mediated by the surface adsorbates remaining on the surface of the dialyzed AuNPs, and it proceeds through a fast charge neutralization reaction in combination with a relatively slow proton generation reaction. This work is important not only for its new insights into the Ag^+^ binding to AuNPs, but also for the experimental strategies that should be useful for probing a wide range of nanoparticle interfacial interactions.

## Author Contributions

SA, XL, and JX all helped with experiment design, performed experiments, analyzed data, plotted graphs, and prepared manuscript. YP conducted Zeta potential measurement. NF supervised the Zeta potential measurement and responsible for part of the writing. DZ designed and supervised the experiments and was responsible for writing the manuscript.

### Conflict of Interest Statement

The authors declare that the research was conducted in the absence of any commercial or financial relationships that could be construed as a potential conflict of interest.
