# Supplementary material for: Surface Plasmon Resonance, Formation Mechanism, and Surface Enhanced Raman Spectroscopy of Ag+-Stained Gold Nanoparticles
Source: Front Chem. 2019 Feb 14;7:27. doi: 10.3389/fchem.2019.00027 (PMC6382679; doi:10.3389/fchem.2019.00027)
Supplement: Supplementary file 1 [file Data_Sheet_1.docx]

Supporting Information

Surface Plasmon Resonance, Formation Mechanism, and Surface enhanced Raman Spectroscopy of Ag^+^-Stained Gold Nanoparticles

*Sumudu Athukorale,^1^ Xue Leng, ^2^ Joanna Xiuzhu Xu,^1^ Y. Randika Perera,^1^ Nicholas Fitzkee,^1^ and Dongmao Zhang, ^1,3,^**

^1^Department of Chemistry, Mississippi State University, Starkville, MS, United States.

*^2^*Department of Chemistry, Chengdu University of Technology, Chengdu, China

*^3^* Department of Chemistry, Xihua University, Chengdu, China.

^*^*Corresponding author: Email:* [*Dongmao@chemistry.msstate.edu*](mailto:Dongmao@chemistry.msstate.edu)

*Fax: 662-325-1618*

Content Page No.

S1. UV-vis spectra for the Ag^+^ treated AuNPs with and without dialysis purification...........S3

S2. Example PRS2 data analysis of AuNPs treated with 150 µM AgNO_3_………..….………S4

S3. The empirical fitting of the kinetic data for the adsorption of Ag^+^ onto

13-nm AuNPs…………………………………………………………………...………..S5

S4. Photograph and UV-vis spectra showing the formation of Ag-citrate salt……………....S6

**S1. UV-vis spectra for the Ag^+^ treated AuNPs with and without dialysis purification.**


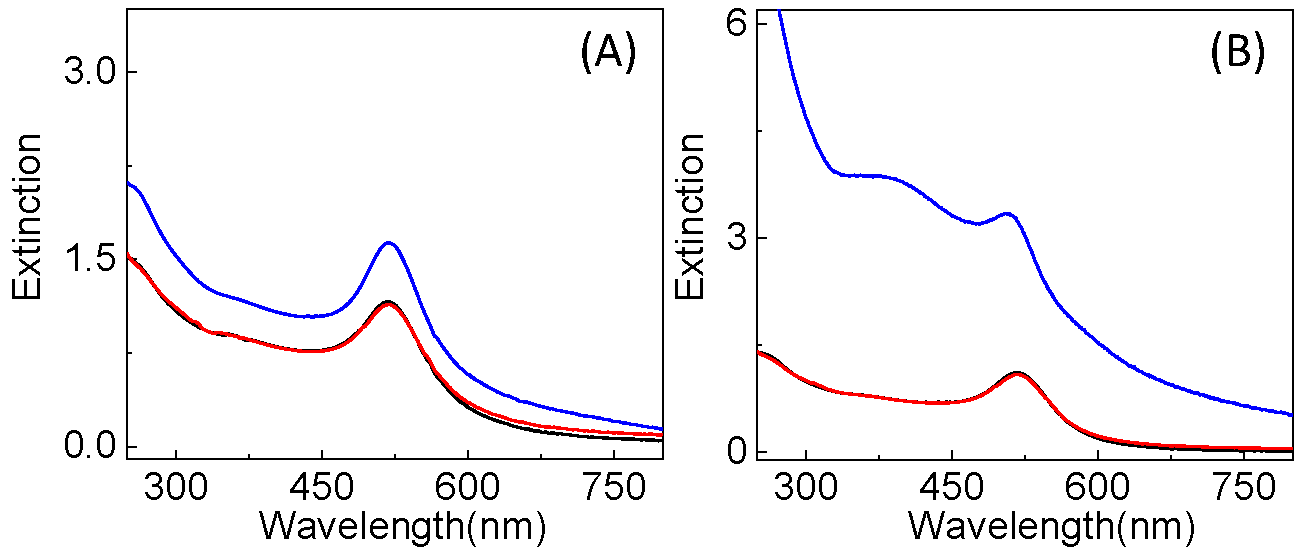


**Figure S1.** UV-vis spectra for the Ag^+^ treated (A) dialyzed and (B) as-synthesized AuNPs. (black) AuNP control spectrum (red) AuNP/KNO_3_ control spectrum (blue) AuNP/AgNO_3_ spectrum. The nominal concentration of KNO_3_ and AgNO_3_ is 2.5 mM.

**S2. Example PRS2 data analysis of AuNPs treated with 150 µM AgNO3**

**Figure S2**. (A) UV-vis extinction spectrum of AuNPs treated with 150 µM AgNO_3._ (B) As-acquired PRS2 solution spectra with excitation and emission polarization combination of (red) VV and (black) VH. (C) Solvent PRS2 VV and VH spectra. (D) Inner-filter-effect corrected PRS2 spectra of the (B) as-acquired spectra using its (A) UV-vis spectra. (D) AuNP specific PRS2 spectra obtained by subtracting (C) solvent background spectra from (C) IFE corrected spectra. (E) G factor spectrum of the spectrofluorometer used for spectral acquisitions. (F) Scattering depolarization spectrum of AuNP (G) Scattering cross-section spectrum of PSNP used as the external reference for the analyte cross-section quantification. The inset is the UV-vis extinction spectrum of PSNP that used to calculate its scattering cross-sections. (H) PRS2 spectra of PSNP that shows PSNP has a depolarization of 0. (I) (red) Extinction cross-section spectrum and (black) absorption cross-section spectrum of AuNP quantified by subtracting the scattering cross-section spectrum from its extinction cross-section spectrum. (J) Scattering cross-section spectra of AuNP calculated with equation $\sigma_{AuNP}^{Sca}(\lambda)=\frac{\left( 1+2P_{AuNP}^{Sca}\left( \lambda\right) \right)}{\left( 1+2P_{PSNP}^{Sca}\left( \lambda\right) \right)}\frac{C_{PSNP}I_{AuNP,VV}^{Sca}\left( \lambda,W \right)}{C_{AuNP}I_{PSNP,VV}^{Sca}\left( \lambda,W \right)}\sigma_{PSNP}^{Sca}(\lambda)$. (K) Scattering-to-extinction spectrum (S/E) of AuNP calculated by dividing scattering cross-section spectrum by its extinction cross-section spectrum.

**S3. The empirical fitting of the kinetic data for the adsorption of Ag^+^ onto 13-nm AuNPs.**


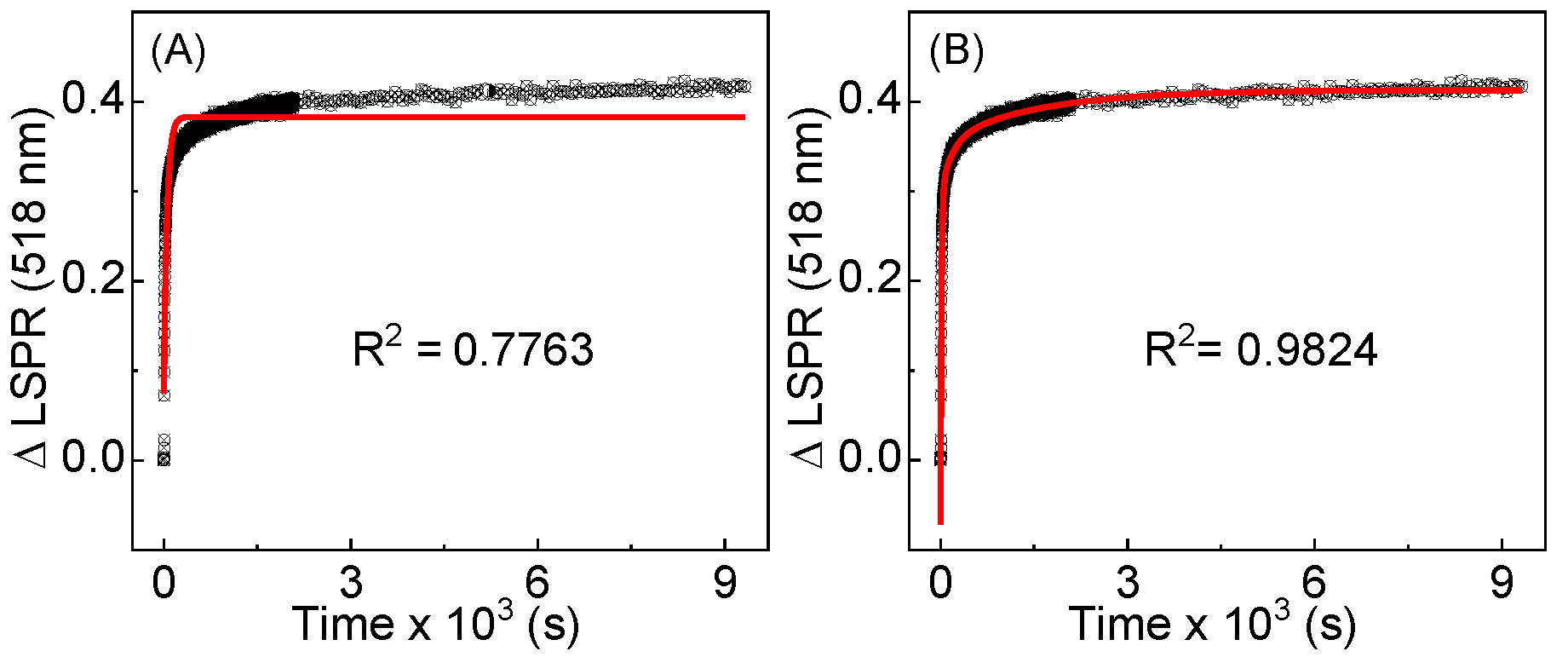


**Figure S3.** Empirical fitting of the time-course of the time-resolved UV-vis spectra with (A) one-pseudo-first-order kinetics ($M=\Gamma_{1}\left( 1-exp\left( -t/{}_{1} \right) \right)$) (B) two-pseudo-first-order equation ($M=\Gamma_{1}\left( 1-exp\left( -t/{}_{1} \right) \right)+\Gamma_{2}\left( 1-exp\left( -t/{}_{2} \right) \right)$). The nominal concentration of Ag^+^ is 100 µM.

**S4. Photograph and UV-vis spectra showing the formation of Ag-citrate salt.**


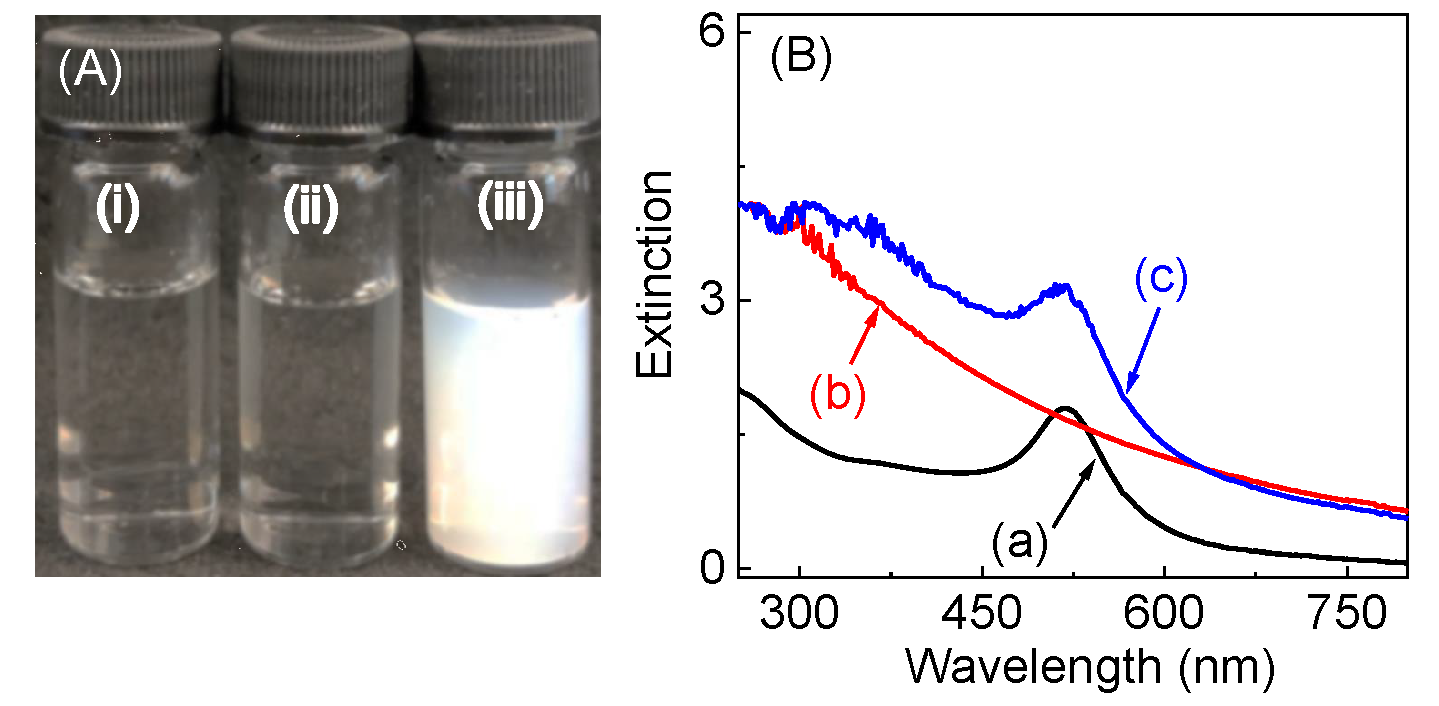


**Figure S4.** (A) Photograph of (i) citrate solution, (ii) KNO_3_/citrate, (iii) AgNO_3_/citrate. Nominal concentration of citrate, KNO_3_, and AgNO_3_ are 4 mM, 12 mM and 12 mM respectively. (B) UV-vis spectra obtained for (a) dialyzed AuNPs/AgNO_3_, (b) as-synthesized AuNP supernatant/AgNO_3_. (c) (as-synthesized AuNP supernatant/AgNO_3_)/dialyzed AuNPs. Nominal concentration of AgNO_3_ is 2.5 mM. The shape of the spectrum indicates that there are insoluble particles formed by Ag^+^ reacting with excess citrate and chloride in the AuNP centrifugation supernatant.
